# The New Masterpiece

**Published:** 1869-11

**Authors:** 


### THE NEW MASTERPIECE.

"Fur," said the Deacon, "'tis mighty plain
That the weakest place must stan' the strain
So the way to fix it, I maintain,
Is only jest
To make that place uz stong uz the rest."

And the Deacon's rule applies as well to artificial teeth ; and we were reminded of this by comparing Dr. Samuel S. White's present style of rivets with former styles, and with those now in general use. Those rivets are philosophical models in their way, and their being inserted into the thickest part of the tooth, insures the strongest possible combination of porcelain and platinum.

